# “To not feel fake, it can’t be fake”: co-creation of a harm reduction, peer-delivered, health-system intervention for people who use drugs

**DOI:** 10.1186/s12954-025-01210-2

**Published:** 2025-06-18

**Authors:** J. Deanna Wilson, Stephanie P. Klipp, Kelsey Leon, Jane M. Liebschutz, Jessica Merlin, Cristina Murray-Krezan, Sommer Nolette, Kristina T. Phillips, Michael Stein, Nate Weinstock, Megan Hamm

**Affiliations:** 1 Department of Family Medicine and Community Health, Perelman School of Medicine, University of Pennsylvania, 51 N 39th Street, Philadelphia, PA 19104 USA; 2 Prevention Point, Philadelphia, PA USA; 3 Division of General Internal Medicine, Center for Research On Healthcare, University of Pittsburgh, Pittsburgh, PA USA; 4 Pregnancy Recovery Center, UPMC, Pittsburgh, PA USA; 5 Center for Integrated Health Care Research, Kaiser Permanente Hawaii, Honolulu, HI USA; 6 Department of Health Systems Science, Kaiser Permanente Bernard J. Tyson School of Medicine, Pasadena, CA USA; 7 Department of Health Law, Policy & Management, Boston University School of Public Health, Boston, MA USA; 8 Center for Biostatistics and Qualitative Methodology, University of Pittsburgh, Pittsburgh, PA USA

## Abstract

**Background:**

People who use drugs (PWUD) continue to experience not only high overdose rates but also growing infectious complications. In response, there has been a growing focus on increasing access to harm reduction resources, particularly among hospitalized PWUD. However, there is limited data on how best to integrate harm reduction into hospital settings. We describe using a Design Sprint, a human-centered design process, to co-create an intervention with people who have lived experience (PWLE) focused on improving access and adoption of harm reduction behaviors for hospitalized PWUD.

**Methods:**

We recruited a sample of PWLE from Pittsburgh, Pennsylvania. We recruited a total of 14 participants over a 3-week period from March to April 2024. There were four Design Sprint sessions, two-hours in length, delivered via HIPAA-compliant zoom. Participants identified intervention components, sketched the intervention, and prototyped the planned intervention process. Sessions were recorded and transcribed verbatim. The team identified intervention components and key themes using thematic analysis.

**Results:**

There were 14 PWLE (mean age 40.4 years; majority white) who participated in at least one Design Sprint session. Participants conceptualized an intervention delivered by a THRIVE navigator who establishes rapport, identifies what if any goals the participant may have, offers information from a menu of harm reduction topics, and helps participants create a Wellness Plan focused on achieving their goals and overcoming likely barriers. The THRIVE navigator will then follow-up via weekly text messages. There were four additional themes that informed intervention content and implementation. These were related to the hospital being experienced as a hostile environment to PWUD; the value of health information being delivered by PWLE who can speak authentically; the importance of creating a flexible participant-led intervention offering a range of content; and the importance of neutrality to building authenticity and attaining participant buy-in.

**Conclusions:**

The Design Sprint process allowed for rich input from PWLE on the design, scope, content, and implementation of the THRIVE intervention. Findings highlight the importance of a peer navigator role to embody relational harm reduction and guide THRIVE participants in education and goal setting around a host of wellness-related behaviors.

## Background

In the setting of escalating overdose deaths [1], we urgently need interventions that address opioid-related morbidity and mortality. While historical approaches to addressing the overdose crisis have focused on increasing access to opioid treatment, we need novel strategies that focus on reducing the burden of morbidity and mortality among those with ongoing opioid use. People who use drugs (PWUD), particularly those with injection drug use have high overdose risk, but are also experiencing rising rates of viral and bacterial infectious complications [2, 3]. In light of this, there has been growing interest in harm reduction as a vehicle to reduce substance use consequences [4–6].

Harm reduction is, at its origins, community-derived [7–10] and harnesses the knowledge and skills of PWUD to improve health for PWUD by reducing substance-use related harms [11]. However, harm reduction opportunities remain underutilized [10] with disparities in harm reduction access among racially minoritized individuals, youth, and in rural communities [12–15]. As PWUD have high rates of hospital contact [16, 17], integrating harm reduction into the hospital setting can serve to increase access equitably [4]. Unfortunately, hospitalization is also often a time where PWUD experience significant addiction-related stigma that may deter patients from seeking care [18–21], contributes to premature patient-directed discharges, and ultimately can lead to poor health outcomes [4, 22–24]. There is a critical gap in how best to reduce stigmatization in people who use substances in medical settings [4, 25].

In contrast to traditional research paradigms that design intervention or programs without soliciting advice from those who they are meant to serve, we recognize the centrality of designing research that includes the voices and opinions of PWUD [26]. PWUD have unique knowledge of local communities and greater understanding of substance use and its context that are valuable to research design and implementation [27, 28]. Designing research with people who have lived experience (PWLE) has been shown to lead to a more diverse recruitment of participants, improved study retention [29–31], and greater likelihood of research leading to changes in policy and practice [32–34]. While most attempts to engage PWLE involve roles in an limited advisory capacity [27, 28], we describe a human centered design process to co-create an intervention with PWLE, entitled Teaching Harm Reduction In Vulnerable Environments, THRIVE, that focuses on delivering harm reduction services to hospitalized PWUD. 

## Methods

### Recruitment

We recruited a sample of PWLE from Pittsburgh, Pennsylvania using local networks of PWLE and partnered with a local harm reduction organization. Participants were eligible if they had lived experience with drug use (e.g. if they endorsed historical or current use of any drugs) and were able to speak and understand English. They were eligible if over the age of 18. We recruited a total of 14 participants over a 3-week period from March to April 2024.

### Design sprint process

The Design Sprint process is a human-centered design approach, developed by Google Ventures [35, 36], that allows for rapid design and testing of models co-created with end-users, in this case PWLE. Previous studies have successfully utilized Design Sprints to improve care in nutrition, diabetes, and pain management in those with end-stage kidney disease [35–37]. The Design Sprint is typically organized around a series of five consecutive six to eight-hour days focused on a series of activities to define the research question and then develop, iterate, and test possible solutions driven by end-users [35, 36]. While typical approaches allow for maximal engagement by end-users, it requires a substantial time commitment that was not feasible for our participant population.

We adapted the Design Sprint process to better align with our research goals and centered population. To allow for the greatest participation, we conducted the sprints via HIPAA-compliant Zoom. Drawing on the previous experiences of team members [37], we opted to use PowerPoint slides with a note taker, a document template emailed to participants, and low-tech tools available through the Zoom interface to capture participant feedback and minimize technological challenges. Recognizing the challenges of engaging participants electronically, we planned for a series of 4 sessions delivered over a three-week period. Participants were asked to participate in at least two of the four sessions. Each session was two hours long and included activities that were adapted to support participants with varying levels of technology and health literacy. Additionally, as the planned intervention was to be tested in a pilot, we concluded the Design Sprint short of user testing with participants identifying intervention components, sketching the intervention, and prototyping the planned intervention process.

### Design sprint implementation

Each day of the Design Sprint followed a similar schedule: The session opened with an overview of housekeeping items (e.g. logistics around compensation), a review of ground rules for the group (e.g. confidentiality and participation), followed by a review of the agenda for the day, and an overview of the previous session findings (if applicable). During the first day of the sprint, participants were presented with findings from prior qualitative interviews of PWUD and providers who work in the healthcare system (conducted by the study team) that highlighted key barriers and facilitators to providing care to PWUD. Participants responded with their own impressions and experiences to more fully describe the current state of care and harm reduction offered to PWUD in hospital-based settings. They then were presented with a rough schematic for a hospital-based intervention to deliver harm reduction services to PWUD delivered by a PWLE using a combination of face-to-face interactions and text messages. Through anonymous polling, participants voted on the acceptability of this skeleton of the intervention. They then participated in a storyboarding exercise to discuss how such an intervention might be delivered and to further tease out the steps of the intervention process. These areas were the primary focus of work in Days 2 through 4. Sessions began with a preliminary overview, after which participants were broken into small groups focused on “remixing and improving” intervention components. Their findings were reported out from their smaller group to the larger group for feedback and further refinement. At the conclusion of the Design Sprint, participants were presented with an overview of the final skeleton intervention to be further refined through pilot testing. Participants were compensated $125 per hour up to a maximum compensation per participant of $750.

### Data collection and analysis

We recorded each session using Zoom. During and after each session, study team members took notes that were reviewed for analysis. Each session was transcribed verbatim. Three team members wrote high-level summaries of each session incorporating notes, video recordings, and transcripts. The primary analyst reviewed the recordings and notes, listened to recordings, and independently identified themes. The secondary analyst then reviewed the primary analyst’s notes, listened to audio recordings, reviewed memos, and built upon the primary analyst’s notes. The third analyst completed a similar process. Analysts met to adjudicate differences and refine the themes using tenets of thematic analysis [38].

## Results

There were 14 PWLE who participated in at least one Design Sprint session. One participant participated in 1 session, three participated in 2 sessions, and nine participants participated in 3 or 4 sessions. The mean age of participants was 40.4 years (ranging from 30–67 years of age). There were 7 women, 6 men, and 1 nonbinary person. 10 participants were White, 1 was Latine, 2 were Black, and 1 did not disclose their race/ethnicity.

**Fig. 1:**
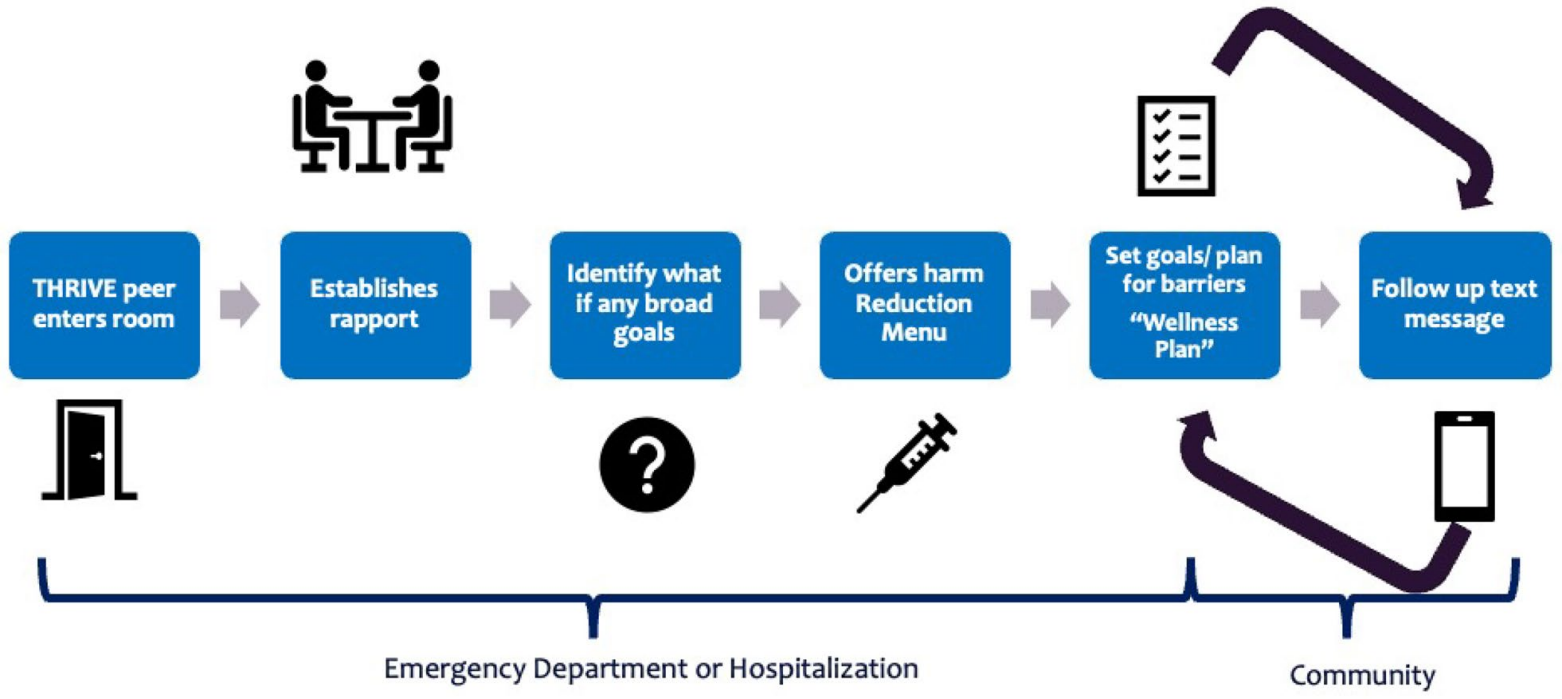
THRIVE: Teaching Harm Reduction in Vulnerable Environments Intervention. The THRIVE intervention consists of a THRIVE peer navigator, a person with lived experience, who meets participants who use drugs early in their stay in the emergency department or hospitalization. The THRIVE peer focuses on establishing rapport, will use person-centered interviewing strategies to identify what if any goals the participant may have, will offer information or resources from a menu of harm reduction topics, and will then help participants create a Wellness Plan focused on achieving the goals they identified as priorities with steps to overcome any potential or likely barriers. The peer navigator will then follow-up with the participant in the community via text message at weekly intervals to follow-up their progress. The follow-up period will be over 12 weeks

Participants conceptualized an intervention that is delivered by a THRIVE peer navigator (a PWLE). The THRIVE navigator meets participants early in the emergency department or hospital stay to establish rapport, identify what if any goals the participant may have, offer information or resources from a menu of harm reduction topics, and help participants create a Wellness Plan focused on achieving the goals they identified as priorities. The peer navigator will then follow-up with the participant in the community via text message at weekly intervals to follow their progress (Fig. 1). Table 1 summarizes findings from the Design Sprint sessions synthesized into specific intervention components.

**Table 1:** Intervention components identified from Design Sprint sessions with emblematic quotes and examples of how it will be operationalized as part of the intervention

Intervention component/strategy	Emblematic quote	How operationalized in intervention
Engage with participants as soon as possible	“[I]t’s really hard to backtrack or to change the outcome of [mistreatment early in the hospital stay] and it’s really frustrating to be quite honest.” Participant A10	Contact participants who may be eligible during the Emergency Department stay if possible
Counter negative stigma	“Applaud people for what they’re doing right, such as coming in the hospital and caring about their health.” Participant A1 “I tell people they *are* in recovery. But guess what you already are in recovery, you’re standing here being proactive about your health, you know, you’re doing everything, you know, I see the people that stand in the line [at the syringe exchange], [they]are the ones that are proactive about their health. And that should be applauded…Because there should be no shame in what they’re doing.” Participant A1	Acknowledge challenges participants experienced to get to hospital, and support their decision-making
Peer navigator as delivery model	“It’s just in the back of my mind, if there’s a way to have that peer engaged throughout the process, so that if, if that initial contact is made, that peer is able to be there with them. … But just having that person with them that can be that that safe space for them.” Participant A11	Utilize a person with lived experience as the vehicle to deliver the THRIVE intervention face-to-face
Non-structured and flexible intervention	“I think once this person is like, saying they were ready to engage, I think the next thing is to like, Okay, what do you need? Like, what? What, like, what are your needs? Like? What are your thoughts right now? […] What, what direction are you trying to go? You know, and then go from there.” Participant A9	Create a flexible series of openings and semi-structured guide for peer interventionalists
Participant led	“Just asking that person, just like, straightforward, like, what are your needs right now? … What, what direction are you trying to go? You know, and then go from there.” Participant A9	Utilize open-ended questions to guide discussions
Offer to teach how participants prefer to learn material	“It’s nice to ask somebody how they like to learn. And if the, you’re able to provide the different type of teaching to them, and whatever capacity that person wants to learn, it’s nice to offer them, you know, different, different ability to learn and video or, you know, pamphlet form.” Participant A8	Open with questions about participant preferences of not only of topics, but ways to learn them. Train peers to deliver information using multimodal approaches
Use one-on-one demonstration	“And doing those demonstrations one on one, I feel like that’s how I’ve had the best and teaching it with people and getting that face to face. And making sure that that … navigators are well-versed in, like all these harm reduction strategies, and they actually know how to demonstrate and teach all these things.” Participant A8	Train peers to offer one–one didactics or demonstrations
Teach back	“I think a lot of times it’s easy to do teach back one on one with individuals, and get a sense of doing that face to face interaction, having those one on one discussions with them. Participant A8	Integrate teach back as a core strategy
Offer variety of harm reduction topics	“Resources that would allow them to begin the recovery process or make their active addiction less stressful. … like a comprehensive document of resources, so they could, we could help them figure out where they could go or where could assist them.” Participant A2	Create content on variety of core harm reduction topics and offer a written menu to participants to prompt discussion
Offer harm reduction supplies	“And I think like you have safer smoking. So that would include like the smoking kits. I’m assuming like the things like that … the injecting more safely or safe use and include the things that would come with that.” Participant A11	Include information and related supplies (e.g. cookers and tourniquets) to go along with related harm reduction topics
Be prepared to address concrete barriers driven by social determinants of health	“Social determinants of health, again, can that person if they have a goal to get their syringes exchanged, can they get to that syringe exchange? So, they can they get to the van sites. Like it’s [Location] Can they get to the van sites? Do they have access to those things? Do they have transportation? What are some of the barriers for them to meet that goal for them?.” Participant A8	Be prepared to address barriers to patient-identified goals, including managing social determinants of health (e.g. transportation, food insecurity, housing insecurity)
Text messaging for follow-up	“12 weeks of text messages or something, which we decided was a long time to text somebody. And we thought maybe four weeks would be okay to like re-evaluate, if it’s a weekly message, you know, if they are already connected, wait, and they aren’t connected with other resources that they initially expressed interest in, maybe could ask if there any barriers they are experiencing.” Participant A17	12 weeks of weekly text messages with check-ins at 4 weeks and 8 weeks
Utilize open-ended messages	“But like, if you’re asking someone checking in on a goal, and let’s say they didn’t do anything, you know, I would want to be careful, like, because like, if they are something like me. And my goal, and I didn’t do it, I probably just wouldn’t respond. … [H]ow to creatively like check in with someone without making them feel bad if they didn’t do it.” Participant A9	Utilize open-ended text messages to carefully check on progress
Check-in frequency	“Getting some kind of approximation about establishing what level of contact would be most comfortable and beneficial for the individual. And then having that pre-established time point of a follow up, and then reevaluate what contact looks like going forward, because people are going to be at different stages of their development and needs and interests.” Participant A5	Create a written timeline and schedule of peer contact
Resources for intervention in multiple formats	“Resources available to them, like on a sheet of paper, you know, that they could take with them and take the time to look over and then go over together.” Participant A2 “I think a level of redundancy in this case is definitely a welcome thing, right? A lot of people have different things going on. So as many avenues as possible to not be over encumbered.” Participant A5	Offer concrete information or resources on paper handouts or digitally
Offer content via text, but not via digital or video links	“Because we had talked about link being sent in text can sometimes be suspect, people don’t love it.” Participant A5 “We’re kind of thinking maybe like, some sort of message board or forum or something where maybe instead of the peers, so instead of like the peers, probing for information to decide what would be relevant for each person, if maybe the peers could just upload different things to this forum, say, for example, and it could be maybe categorized, or sorted by neighborhood or something like that.” Participant A17	Create an online repository of harm reduction education, digital pamphlets, and map to local services
Support THRIVE peers with supervision and resources	“I think being that Thrive peer, being well rounded and educated, and experienced and educated themselves, to have them conversations and also know when, if they don’t know how to ask somebody for help themselves too, for that, for the person receiving the services.” Participant A11 “Especially for the peer, making sure that they understand how to set those boundaries, and that they’re abiding by some sort of, you know, code of ethics. … Also, like how to care for themselves? And how are we going to support the peers thinking about like, how do we make sure that there’s support for them in case it’s, you know, there’s some like secondary trauma.” Participant A4	Define scope of peers’ practice, ensure ample resources are available for local referrals, provide supervision/peer group support to navigate setting boundaries and managing workload

There were four themes generated from the Design Sprint process that informed both intervention content and implementation. These were related to the hospital being experienced as a hostile environment to PWUD; the value of health information about drugs being delivered by PWLE who can speak authentically about the topic; the importance of creating a flexible participant-led intervention offering a range of content; and the importance of neutrality and lack of judgement to authenticity and attaining participant buy-in.

Theme 1: Hospital experienced as a hostile environment to people who use drugs.

Participants identified the hospital as a place where enacted or anticipated stigma would likely serve as a barrier to any hospital-based, harm reduction-focused intervention. They describe staff, from security guards to physicians, as propagating stigma and contributing to environments that felt unsafe for PWUD. As participant A9 says: *“Hospitals are never like a safe place ever. …. I generally just assume that it’s going to be a shitty experience”* and also *“[Y]ou never really know if you’re gonna get offered treatment or gonna get arrested.”* Hospitals were viewed as hostile and carceral spaces that often failed to center the needs or well-being of PWUD.

Multiple participants described negative experiences they personally encountered as either PWUD or those they witnessed as PWLE working in hospital environments (e.g. as a nurse or as peer navigator) directed towards other PWUD. Participants described experiences with poorly managed withdrawal or suboptimal medical care and a general feeling that providers lacked compassion and were judgmental to PWUD. Participant A1, who works as a peer navigator in a hospital system, says: *“I’m jaded towards doctors being compassionate, because it’s kind of like with police. That’s why police, they have this own little group of people to talk to victims, because police can’t be compassionate. It’s kind of the same with doctors. That’s why we’re [i.e., peers] here because doctors can’t be fucking compassionate, don’t know how to talk to people.”* Because physicians were viewed as lacking basic skills to communicate empathetically with PWUD, particularly in discussions around active substance use, participants identified the importance of having PWLE to deliver information in a compassionate, nonjudgmental manner.

Participants believed enacted stigma could prime potential PWUD to refuse to engage with the THRIVE study. For example, as participant A10 stated*: “If they have been treated poorly and feel that their withdrawal is not being managed properly, that they’re not being heard, that they don’t feel that there is going to be hope that they are going to be treated well. When someone comes in after the fact, they don’t want to hear it…. [T]hey already made up their mind and so anything that comes after, it’s very hard to, to backtrack that their fight or flight has already started to go off, they’re feeling unsafe and so those, it’s not a time for rational thinking it’s like not a time to be like, okay, well now let’s listen to this person.”*

Theme 2: Health information about using drugs should be delivered by people with lived experience using drugs who can speak authentically about the topic.

Participants identified the importance of having THRIVE interventionists be peer navigators or PWLE who can have authentic and genuine interactions around substance use. In direct contrast to the way participants described members of the healthcare team and the hospital environment, they repeatedly described the role of the THRIVE navigator as creating a “safe space” or building a culture of “safety” for PWUD. Participant A10 describes how their small group conceptualized the peer role: “*The peer has to be authentic. They have to be real. They have to really believe in harm reduction. I think that’s important. They need to believe in all pathways to recovery. The person [study participant] needs to feel safe, they need to feel safe enough to receive those services.”*

Participants describe peers as capable of greater authenticity because they could leverage their own previous experiences using drugs to relate in a genuine way to participants. Participant A4 says: *“Real recognizes real so really making sure that you’re like talking the talk and it’s like we’re different than the medical professional and the rest of the staff. It should feel like a different experience when you’re working with the Thrive peer. We talked about self-disclosure if you have it to disclose, so especially if, if there’s some commonalities within drugs of choice, or experience, if there is recovery experience, and maybe this person has followed a similar path or something, if there’s crossover in experiences being able to self-disclose, if you feel comfortable with doing that, really helps to build that rapport.”* The ability of peers to leverage their lived experience distinguished them from medical members of the team, and that disclosure could potentially facilitate stronger rapport.

Participants believed THRIVE peer navigators needed to feel genuine concern and compassion for THRIVE participants. Authenticity was important not only philosophically, but because it would allow them to be more effective at having honest conversations with participants. As participant A5 says: “*For it, to not feel fake it, it, it can’t be fake. … And I think what if you actually show that you care, and you’re concerned about what they’re going through where they’re at, what brings them into this space that day, they’re probably going to want to tell you about it anyway. Without, without you trying to pull it out of them.”* Authentic concern would lead to greater effectiveness in gathering information from participants and staying connected with them over a longer period. As Participant A4 says: “*Building rapport keeps their trust. So, it’s more likely that they’re going to come back to us if they’re back in the hospital, or maybe keeping in touch through the text messaging and things like that… the most important thing is building rapport and building that relationship. So, it kind of, everything kind of rests on that interaction.”*

Theme 3: The intervention must be flexible, participant led, and include a wide variety of content.

Design Sprint participants discussed the varied experiences and desires individual THRIVE study participants may hold. As Participant A4 says: “*So just really kind of being mindful that everyone’s coming to the table with a different, from a different place.”* For them it highlighted the importance of having a peer-delivered intervention be flexible and responsive to a participant’s needs. Participants stressed the importance of drawing on their own lived experience to help THRIVE participants feel comfortable sharing what, if any, their goals were. For example, Participant A2 says: *“Like yes, I’ve been in a point where I did not want help. And I want you to know that this is the place where you can come. Whatever the circumstances using, not using. Know whatever your goal is on we’re going to work on them.”* THRIVE participants should be freed from the burden of feeling they needed to focus on recovery-oriented goals and instead be able to share whatever goals felt most resonant for them at the moment. Design Sprint participants identified a wide scope of content that should be available for THRIVE participants: overdose prevention, including naloxone; wound care, including information on xylazine wounds; safer consumption (smoking and injecting), medications to treat opioid use disorder, drug testing, preventing HIV, booty bumping, coping with stress, and ways to link to care.

Participants describe several relational approaches, and also concrete strategies to structure THRIVE navigator conversations to facilitate participant-led discussions. Table 2 summarizes Design Sprint recommendations for *how* peers can build genuine rapport with THRIVE participants (Table 2).

**Table 2:** Recommendations for Strategies and Approaches to Be Used by THRIVE Peer

Peer strategies	Emblematic quote
Respond with empathy	“The best thing we can do is respond with empathy, empathize with them. Let them know that you’re, you know, here for them when they’re ready. I guess just, I mean, because we don’t want to make them uncomfortable. At the end of the day, peer services are supposed to be voluntary. And we’re here to help when they’re ready.” Participant A9
Leverage lived experience to create safe space	“I just kind of like, do the shared lived experience thing? Like yes, I’ve, I’ve been in a point where I did not want to help. And I want you to know that this is the place where you can come. Whatever the circumstances using not using, know whatever your goal is on we’re going to work on them.” Participant A2 “Creating a safe space for the individual to build a relationship and [A12] talked about, you know, using your lived experience and sharing your own story to do that.” Participant 10
Normalize all pathways/needs are valid, explicitly do not value abstinence over other goals/desires	“I’m there to support them unconditionally, that I do not have expectations for them, you know, that I am there to help them with whatever I can. So I’m very, I plainly state to why I’m there and letting them know that I don’t have expectations for them to enter into treatment to receive my services or whatever, like, I am just here to help you make that so your experience in the hospital, I’m there to help make this process of being in the hospital as easy as possible, so that you can get the treatment that you deserve and need.” Participant A10 “Having like, their goal may just be to take a shower that day, it may not be to make a dentist appointment, or, or follow through with where we feel, you know, we say this a lot like service, like services look different to people, to each person, like, we’re out here providing a service, and that service may just be a toothbrush, it may not be connected to a treatment facility.” Participant A11
Focus on immediate needs	“What are your immediate needs? Does that look like, action to safe using does that look like connection to take home wound care kits? Does that look like education on how to care for that, you know, for your partner out there.” Participant A9
Offer information on healthy coping and problem-solving skills	“Healthy coping and problem-solving skills. So, helping them with problem solving, helping them maybe see better coping skills and things like that.” Participant A10
Empower patients through messages emphasizing they deserve treatment and basic needs regardless of a decision to use drugs	“We talked about empowerment, in really empowering them and maybe providing some in a way of saying like, you deserve treatment and you deserve healthcare, you deserve housing, you deserve everything everyone else deserves whether or not you use drugs or stop using drugs like so that like really, I feel like I’ve met a lot of people and individuals who are sort of beat down by our system and our health care and people and they really don’t feel deserving of treatment, because of the fact that they’re going to continue to use drugs. And so I feel like almost educating them like about harm reduction is important in a way, because a lot of people don’t know that it exists or that mindset exists that people deserve to get housing before, they need to stop using drugs like that maybe somebody needs stable housing and have had an unhealthy environment like for them and their mental health and everything else, to stop using drugs.” Participant A10

Theme 4: Neutrality and lack of judgement are crucial to authenticity and attaining buy-in.

Participants discussed the importance of messaging of the intervention itself and the intervention components to obtain the greatest buy-in from PWUD. For example, while the team discussed the importance of having a person-led intervention, the majority of participants balked at motivational interviewing as the way in which individual motivations were solicited as it often felt “*fake*” to participants. They interpreted it as providers “*moving you towards a goal they have in mind that is not necessarily in your interest*.” Participants described the process as *“playing a game to make you think it’s your idea.”* Participant A4 says: “*I get a little triggered by motivational interviewing… it just feels like the ultimate goal is always going to be abstinence, or whatever sobriety or whatever. So even just assessing someone’s motivation, like motivation for why and planting that seed, when I’m on the other side of it, as a person who uses drugs, like I automatically just feel shamed.”* While participants supported the basic tenets of motivational interviewing (patient-led discussions around patient-identified goals), their experience had been that these conversations were rarely genuine and often had a hidden agenda. To be explicit in countering this, the THRIVE intervention has to explicitly value any patient-derived goals, including those that involve ongoing use.

Similarly participants had visceral reactions to asking THRIVE participants to focus on goals related to being healthy as it implies their current state or method of use is unhealthy. As Participant A4 says: “*I also don’t love the word healthy, because what is healthy? Who decides what is healthy? So, I think asking … if using the word, well-being is better, or overall well-being or something. But, I just, yeah, the word healthy never really feels good when somebody asks me that, especially as a person who not only has used drugs, but I’m also in a fat body.”* Participants believed to truly embrace a harm reduction philosophy, the THRIVE intervention needs to engage participants in a way that is explicitly value neutral. One participant (A2) shared they discussed in their small group, that THRIVE should have participants make a “*wellness plan*” or a “*thriving plan*” that focused on whatever goals they could identify as important or of value to them.

While some of the language that participants bristled at was viewed as problematic as it prioritized abstinence, other phrases were seen as problematic because they were patronizing. For example, Participant A17 describes taking offense at being offered education on substance use but was open to other ways of framing the discussion. “[*O]ffering education is to me is sort of, it could be insulting. I’m just thinking for myself all the time I’ve spent in the hospital. When I was using and, and if people are well, oh, do you need some education [on using drugs]? I’m like, no, I know everything,* you *need some education. Resources are great. If somebody is like, do you want some resources? I’m always like, yes, I need more resources. You know, it’s just a very positive word. And information too. Information. Resources.”* Similarly, participants discussed strong preferences for receiving information via flyers over pamphlets, which were viewed as more medical or sterile documents.

## Discussion

The Design Sprint methodology provided a useful approach for co-creating a hospital-based harm reduction intervention with PWLE. The participatory methods allowed for rich input from PWLE on the design, scope, content, and implementation of the THRIVE intervention. Participants identified the hospital environment as a hostile setting to PWUD, and they welcomed an intervention that authentically engaged PWUD regardless of their desires to stop or continue substance use. They described the necessity of any harm reduction-focused intervention to explicitly counter stigma in its design and implementation. Participants repeatedly highlighted the need for harm reduction interventions in the hospital space to be highly relational, driven by strong rapport with a PWLE serving as study interventionalist as a means to counter stigma.

Design Sprint participants noted the value of having a PWLE deliver THRIVE intervention content in a peer navigator role. They highlighted the value of having a PWLE as they were particularly suited to have authentic conversations with THRIVE participants about substance use, but also because they believed these persons would produce a more effective intervention. The focus on the peer or PWLE as the ideal vehicle for delivering intervention content aligns with a robust body of literature highlighting the important role for PWLE in the delivery of harm reduction services [39–41]. Several studies show that not only do PWLE improve feasibility, acceptability, and quality of harm reduction services offered to PWUD [42], but also can leverage their own lived experience, they are often better able to facilitate trust-building, support engagement, and increase credibility [40, 43].

The THRIVE intervention will recruit participants in the acute hospital setting, an environment described by Design Sprint participants and multiple qualitative studies as being stigmatizing and threatening to PWUD [19, 44, 45]. Studies have shown that PWLE are able to help minimize enacted stigma [46, 47], create safe environments with less differentials in power [46, 47], and improve outreach to populations less likely to engage in care [48, 49]. Research has also demonstrated that intervention in emergency department after an adverse medical event can be an opportune moment to engage PWUD with harm reduction resources [50, 51]. Additionally, studies have shown they may be more effective in knowledge transfer than other team members based on their ability to leverage lived experience [52].

Consistent with the broader literature that describes harm reduction as a strategy not just focused on supplies or services, but also a way of interacting with patients [10, 25, 53], Design Sprint participants stressed the importance of the THRIVE peer navigator embodying relational harm reduction in the spirit of participant A5’s comment that, “to not feel fake, it can’t be fake”. In contrast to focusing exclusively on strategies that reduce substance-use related harms, relational harm reduction is a broader term that operationalizes core tenets of harm reduction in medical settings; it encompasses a patient-provider relationship that is non-judgmental and respectful of patients’ autonomy [53]. Core tenets of this approach are defined as humanism, pragmatism, individualism, autonomy, incrementalism, and accountability without termination [10, 53]. These tenets align with the peer navigator role as conceptualized by Design Sprint participants. Participants described a shared belief that peer navigators whose practice is informed by these principles would have a greater ability to successfully engage and retain THRIVE participants. Participants repeatedly describe the end-result of these types of actions by peer navigators as creating a veritable “safe space” in the context of a hospital system that most often feels unsafe to PWUD. Their beliefs in the strength of this philosophy are supported by studies showing harm reduction approaches that value low-threshold, pragmatic care [25], patient autonomy, and patient-centered goals are more likely to allow participants to stay engaged when their long-term goals are not abstinence [54], and creates an experience of demarginalization and increased engagement [55].

The peer-led nature of the intervention was not solely relational, but also translated to the participant’s recommendations for how intervention content should be delivered. While Design Sprint participants recognized the constraints of a clinical trial, they still valued as much flexibility with respect to the delivery of content, timing of content delivery, and length of the period of engagement. Participants chafed against the idea of a highly structured or standardized way of delivering intervention components, building rapport, or following up with participants. They highlighted the need for peers to be accessible and to reduce communication barriers by providing cell phones as part of the intervention. Having messaging that was less prescriptive and could respond to the needs of participants was identified as a crucial strategy for authentic engagement. Patient-led goal setting around implementing harm reduction strategies (either accessing services, utilizing supplies, or strategies to explicitly reduce harms) was viewed as a positive approach, but participants emphasized that how the THRIVE navigator followed-up on those goals needed to be done carefully to avoid shaming and potentially alienating those who made limited progress. While multiple studies support delivery of harm reduction services to PWUD in ways that value flexibility and respect patient autonomy [25, 56], operationalizing this approach in a clinical trial protocol presents a challenge. There is an inherent tension between the ideal program conceptualized by Design Sprint participants and that which can be evaluated using the framework of a traditional clinical trial. Additional research studies investigating these kinds of approaches using quasi-experimental research methods and pragmatic clinical trials may better embody participant preferences.

While the study demonstrated an effective strategy to co-create an intervention with PWLE, it has several limitations. We engaged a sample of PWLE from Pittsburgh. Based on the social and cultural context of substance use, the findings from this group may not be generalizable to other areas. We also recognize the importance of intersectionality with respect to both substance use and the experiences of individuals who are hospitalized. Our sample is reflective of Pittsburgh demographics but did not include sufficient diversity in participant perspectives to capture the nuances of experiences and perspectives of different races on the intervention topic. Future studies that are designed to better target racially minoritized and other marginalized populations who use drugs are warranted.

## Conclusions

In summary, we used a Design Sprint process to engage PWLE in the co-creation of the THRIVE intervention, a hospital-based intervention delivering harm reduction services to PWUD. The participatory methods allowed for rich input from PWLE on the design, scope, content, and implementation of the THRIVE intervention. The co-creation process underscored the importance of a peer navigator role to embody relational harm reduction and guide THRIVE study participants in education and goal setting around a host of wellness-related behaviors. The co-creation process with PWLE suggests greater potential relevance and efficacy of the proposed intervention. A clinical trial is planned that will test both the effectiveness of the intervention in changing behaviors among PWUD recruited from hospital settings.

## Data Availability

Design Sprint transcripts are available upon request unless where it would otherwise compromise individual privacy, and with prior agreement by study authors.

## Abbreviations

PWUD: People who use drugs
PWLE: People with lived experience
THRIVE: Teaching Harm Reduction in Vulnerable Environments
